# Leadership Perceptions, Educational Struggles and Barriers, and Effective Modalities for Teaching Vertigo and the HINTS Exam: A National Survey of Emergency Medicine Residency Program Directors

**DOI:** 10.5811/westjem.20787

**Published:** 2024-12-31

**Authors:** Mary McLean, Justin Stowens, Ryan Barnicle, Negar Mafi, Kaushal Shah

**Affiliations:** * AdventHealth East Orlando, Department of Emergency Medicine, Orlando, Florida; † ChristianaCare Health System, Department of Emergency Medicine, Newark, Delaware; ‡ The Warren Alpert Medical School of Brown University, Department of Emergency Medicine, Providence, Rhode Island; § San Joaquin General Hospital, Department of Emergency Medicine, French Camp, California; ∥ Weill Cornell Medicine, Department of Emergency Medicine, New York, New York

## Abstract

**Introduction:**

The utility of the three-part bedside oculomotor exam HINTS (head impulse test, nystagmus, test of skew) in the hands of emergency physicians remains under debate despite being supported by the most recent literature. Educators historically lack consensus on how specifically to teach this skill to emergency medicine (EM) residents, and it is unknown whether and how EM residency programs have begun to implement HINTS training into their curricula. We aimed to characterize the state of HINTS education in EM residency and develop a needs assessment.

**Methods:**

In this cross-sectional study, we administered a survey to EM residency directors, the themes of which centered around HINTS education perceptions, practices, resources, and needs. We analyzed Likert scales with means and 95% confidence intervals for normally distributed data, and with medians and interquartile ranges for non-normally distributed data. Frequency distributions, means, and standard deviations were used in all other analyses.

**Results:**

Of 250 eligible participants, 201 (80.4%) responded and consented. Of the 192 respondents providing usable data, 149/191 (78.0%) believed the HINTS exam is valuable to teach; 124/192 (64.6%) reported HINTS educational offerings in conference; and 148/192 (77.1%) reported clinical bedside teaching by faculty. The most-effective educational modalities were clinical bedside teaching, online videos, and simulation. Subtopic teaching struggles with regard to HINTS were head impulse test and test-of-skew conduction and interpretation, selection of the correct patients, and overall HINTS interpretation. Teaching barriers centered around lack of faculty expertise, concern for poor HINTS reproducibility, and lack of resources. Leadership would dedicate a mean of 2.0 hours/year (SD 1.3 hours/year) to implementing a formal, standardized HINTS curriculum.

**Conclusion:**

Despite controversy surrounding the utility of the HINTS exam in EM, most residency directors believe it is important to teach. This needs assessment can guide development of formal educational and simulation curricula focusing on residency directors’ cited HINTS exam educational struggles, barriers, and reported most-effective teaching modalities.

Population Health Research Capsule What do we already know about this issue?

*When properly used, the HINTS exam has high diagnostic accuracy for central causes in dizzy patients, but the state of HINTS education in (EM) is inadequately characterized.*

What was the research question?

*What are program leadership perceptions, educational practices, and barriers to teaching HINTS in EM residencies?*

What was the major finding of the study?

*78.0% of program leaders believe the 3-part oculomotor exam is valuable to teach, and 64.6% offer formal HINTS education sessions.*

How does this improve population health?

*Teaching HINTS to EM residents requires improved curricula, resources, and faculty expertise. Better education may help translate promising HINTS literature into clinical practice.*

## INTRODUCTION

### Background

Posterior stroke presenting with dizziness is misdiagnosed by emergency physicians (EP) in 35% of cases,^1^ which can lead to severe debilitation and sometimes death.^2^ Paradoxically, of patients discharged from the emergency department (ED) with a diagnosis of dizziness or vertigo, only 1 in 500 is diagnosed with a stroke within the first month.^3^ With advances in stroke treatment modalities, it makes sense that there is heightened emphasis on detection. In 2013, the annual cost of imaging for acute dizziness in United States EDs was nearly $4 billion.^4^ Much of this cost is due to utilization of non-contrast computed tomography (CT) of the head^5^ despite its low sensitivity for detecting posterior fossa stroke (mean 41.8%, 95% confidence interval 30.1–54.4%)^6^ and the low lifetime cost-effectiveness compared to magnetic resonance imaging (MRI).^7^ Specifically, it is estimated that over $1 billion per year is wasted on inappropriate CT imaging for patients with dizziness/vertigo.^8^

A worldwide survey of EPs published in 2008 found that the development of a better clinical decision rule for identification of central vertigo was the second highest clinical priority for participants.^9^ Management of dizziness and vertigo is included in the Joint Task Force Emergency Medicine Model of Clinical Practice,^10^ and EPs are expected to diagnose and manage patients with these chief complaints. It is, therefore, incumbent upon EM residency programs to provide adequate education and training on dizziness and vertigo. However, a 2005 study found that only 35% of EM residency programs required a clinical neurology or neurosurgery rotation, and an annual mean of 12 hours (of 280 total didactic education hours) was dedicated to neurologic emergencies.^11^ It is unknown how much of this time is devoted specifically to dizziness and vertigo, or exactly what is being taught regarding appropriate history, physical, and diagnostic workup recommendations.

The clinical HINTS exam (head impulse test, nystagmus, test of skew)^12^ is a three-part bedside oculomotor exam with diagnostic accuracy for central vertigo similar to that of MRI. A 2023 Cochrane Review of 12 studies and 1,890 participants found the clinical HINTS exam to be 94% sensitive and 87% specific.^13^ This exam may be appealing to the EP because it is purported to be a rapid and low-cost bedside evaluation. However, literature suggests that its diagnostic accuracy has fallen short for EPs using the HINTS exam in clinical practice, with findings suggesting that the reasons are application to inappropriate patients (eg, those without acute vestibular syndrome and nystagmus) and difficulty in interpreting head impulse test (HIT) results.^14^ ^,^ ^15^ In addition, the literature has shown poor inter-rater reliability among EPs using the HINTS exam.^16^ With these concerns in mind, two other clinical decision tools have since built on HINTS principles. The first is the HINTS “plus” tool, which adds a hearing test (95.3% sensitive and 72.9% specific).^13^ The second is the STANDING (spontaneous nystagmus, direction, head impulse test) algorithm, which uses two parts of the HINTS exam and additional physical exam maneuvers (93–100% sensitive and 72–94% specific).^17^ ^–^ ^19^

The 2023 American College of Emergency Physicians (ACEP) Clinical Practice Guideline offers specific HINTS exam recommendations and cautions: “Before employing a maneuver such as HINTS, physicians should have sufficient education to perform the technique; not using tools such as HINTS may lead to excessive testing and admission; and incorrect implementation may lead to an increased risk of misdiagnosis.”^20^ In addition to ACEP’s recommendations, in 2023,the Society for Academic Emergency Medicine released Guidelines for Reasonable and Appropriate Care in the Emergency Department (GRACE-3): Acute Dizziness and Vertigo in the Emergency Department. They had similar recommendations that EP education should involve the following : “receive training in the HINTS exam; use the HINTS exam (once properly trained) in patients with nystagmus; and consider the HINTS exam as the first-line test over MRI (if a HINTS-trained clinician is available).”^21^ ^,^ ^22^ The authors of GRACE-3 also acknowledged a discordance in that most EPs have not received special training in the use of the HINTS exam. This lack of special training may have led to the HINTS testing inaccuracies reported in the recent literature.^14^ ^,^ ^15^ This begs the question of which, if any, educational tactics have been effective.

From the recent GRACE-3 guidelines^21^ and releases by EM societies,^10^ ^,^ ^20^ there is a clear call for EM HINTS education and HINTS exam integration into the EM clinical arena. However, the current state of HINTS exam acceptance, education, and training is unclear. If HINTS curricular implementation has occurred, information about the needs, barriers, teaching struggles, and educator perspectives may add further weight to the argument for our specialty’s overall acceptance of the HINTS exam.

### Importance

The standard of care for the ED evaluation of dizzy patients may be evolving to embrace the HINTS exam, but translation of the literature to clinical practice remains unclear. It is also unclear what proportion of EPs have been adequately trained in the use of the HINTS exam. Furthermore, residency programs may lack the faculty expertise, time and funding to add new items such as HINTS education to their curricula. Programs that have adopted the societal guidelines addressing the HINTS exam may have already adjusted their didactic and simulation content. Supporters of the HINTS exam will recognize the importance of a needs assessment with regard to residency efforts and perceived challenges and barriers to dizziness evaluation and HINTS education. Skeptics will find the knowledge of current HINTS teaching paradigms useful to determine their own practice and the potentially evolving standard of care.

### Goals of This Investigation

While recent research supports a need for change in our ED clinical practice, it has yet to be assessed whether these ideas are currently being taught within EM residency programs, and if so, how they are being taught. Our goal in this investigation was to assess the current United States EM residency program leadership perspectives, teaching paradigms, teaching barriers, and future needs for implementing educational curricula on assessment of the dizzy patient, with a particular focus on the HINTS exam. The results of this educational needs assessment can serve to guide and refine the construction of educational resources including didactic and simulation modalities.

## METHODS

### Study Design and Setting

This was a cross-sectional observational study in a virtual setting. Participants were offered no incentives, there was no funding, and the study was institutional review board-approved as exempt. An electronic survey was administered to EM residency directors between April 6–July 13, 2023. The study was conducted in compliance with STROBE (Strengthening the Reporting of Observational studies in Epidemiology) cross-sectional reporting guidelines.^23^

### Selection of Participants

Included were current program directors for categorical EM residency programs in the US. Excluded were program directors from residency programs that received initial accreditation from the Accreditation Council for Graduate Medical Education on or after January 1, 2020. The rationale for this exclusion was that new programs were less likely to have administered an entire educational curriculum cycle. The target population included 250 program leaders (one from each eligible program). Program director contact information was obtained from medical society databases and residency program websites. While both work and personal emails were often publicly available, we prioritized making contact via work emails. See Appendix A for the participant recruitment message.

### Survey Development

The survey instrument was developed, tested, and validated using a rigorous process with close guidance and leadership from seasoned national medical education experts via a formal Medical Education Research Certification program through the Council of Residency Directors in Emergency Medicine. We followed the systematic, seven-step protocol for developing medical education research questionnaires described by Artino et al.^24^ Formal focus groups were used to propose, discuss, and rework survey items using an iterative process until consensus was reached regarding face validity and internal consistency. The survey was piloted by a group of 20 members of the nonprofit medical education alliance ALL NYC EM (consisting of EP medical educators, residency leadership members, and resident education fellows) for feedback on clarity and usability. The sole consensus recommendation was to shorten the survey, which was done prior to national distribution. Final survey items included program/institution demographics and questions about perceptions and practices regarding dizziness, vertigo, and HINTS exam education within each residency program. See Appendix B for a copy of the complete survey tool.

### Study Protocol

We used the electronic platform SurveyMonkey (SurveyMonkey Enterprise, San Mateo, CA) to distribute the survey and collect data. The 250 program directors were initially contacted individually via email with the recruitment message and their personalized survey link. Subsequent contact attempts (required for 235 program directors) were made for non-responses or incomplete surveys. At the end of the data collection period, all complete and partial surveys were included in analysis if the participant provided data beyond the informed consent question. Except for the informed consent question, no survey question was required. This allowed participants to opt out of answering specific questions if they wished while still enabling them to participate. Missing data from participants who opted out of a question was not included in the calculations for subsequent statistical analysis for that item.

### Outcomes

Intended outcomes centered around residency directors’ HINTS exam perceptions as well as current HINTS educational practices within residency programs, resources available, and curricular needs. The purpose of gathering information on these outcomes was to generate a needs assessment for dizziness and HINTS exam curricula in EM residencies.

### Analysis

We analyzed data using R version 4.3.2 for MacOS (R Foundation for Statistical Computing, Vienna, Austria). Likert-scale data was analyzed using medians and interquartile ranges for non-normal data distributions or using means and 95% confidence intervals (CI) for normal data distributions. We tested normality of data distributions by examining estimates of skewness and kurtosis for each scale, as well as by plotting histograms and comparing distributions to the normal curve. Normality was concluded only if all estimates of skewness and kurtosis fell below the thresholds of 2 and 7, respectively, and all histograms aligned closely with the normal curve.^25^ We used the Wilson score statistic for calculation of 95% CIs for binomial proportion items (yes/no items with an answer of “yes” defined as a positive result).^26^ ^,^ ^27^ Frequency distributions were used to analyze questions about struggles and barriers to teaching the HINTS exam. We used descriptive statistics (means and standard deviations for all other quantitative data. As participants were permitted to skip any question, missing data was omitted from item-level analyses. See Appendix C for details on missing data and item-level response rates.

## RESULTS

### Characteristics of Study Subjects

Of 250 eligible programs, leadership from 204 opened the survey and 201 provided informed consent for an overall survey response rate of 80.4%. Among consenting respondents, 192 programs provided useful data beyond the initial informed consent question. See Appendix D for the enrollment flowsheet. Participating program demographic characteristics were well representative of the population of all eligible programs (see Appendix E).

### Main Results

**Figure 1.**
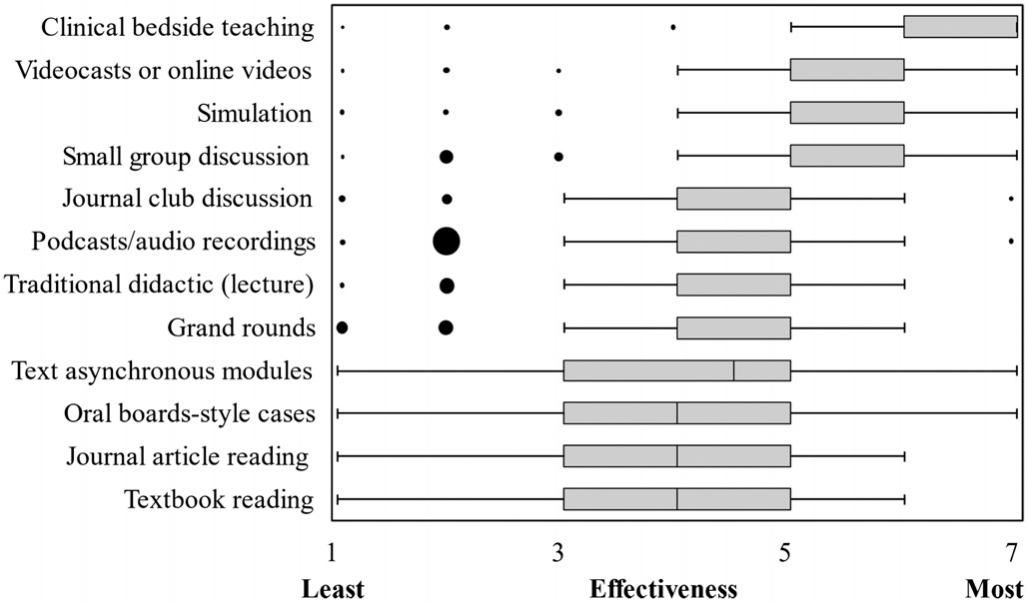
Box-and-whisker plot of leadership-perceived educational modality effectiveness for teaching the HINTS* examination: 188 participants provided usable data on this item (item-level response rate 75.2%). Likert-scale ratings from least (1) to most (7) effective were used. Data for one of the 12 modalities (clinical bedside teaching) was not normally distributed and thus medians and interquartile ranges (IQR) were used for all analyses. Medians are represented by thick vertical lines, IQRs are represented by gray boxes, whiskers represent 1.5* IQR, and outlier data is represented by black dots, with the area of each dot proportional to the answer frequency. HINTS*, head impulse test, nystagmus, test of skew.

Overall, 149/191 (78.0%) believed the HINTS exam is valuable to teach, 16/191 (8.4%) believed it is not, and 25/191 (13.1%) were unsure. On subgroup analyses of these and other key survey items, program demographic factors (program length, setting, type, and region) were of no statistical significance after controlling for multiple comparisons. The most effective educational modalities for teaching the HINTS exam were reported to be clinical bedside teaching, videocasts/online videos, and simulation. Perceptions of modality effectiveness varied widely. See Figure 1.

Program leadership reported perceptions that their residency graduates were, on average, more confident and competent than their faculty members at performance and interpretation of the HINTS exam. They also reported perceptions that, for both residency graduates and faculty members, confidence was higher than competence. However, none of these patterns reached statistical significance. See Appendix F.

**Figure 2.**
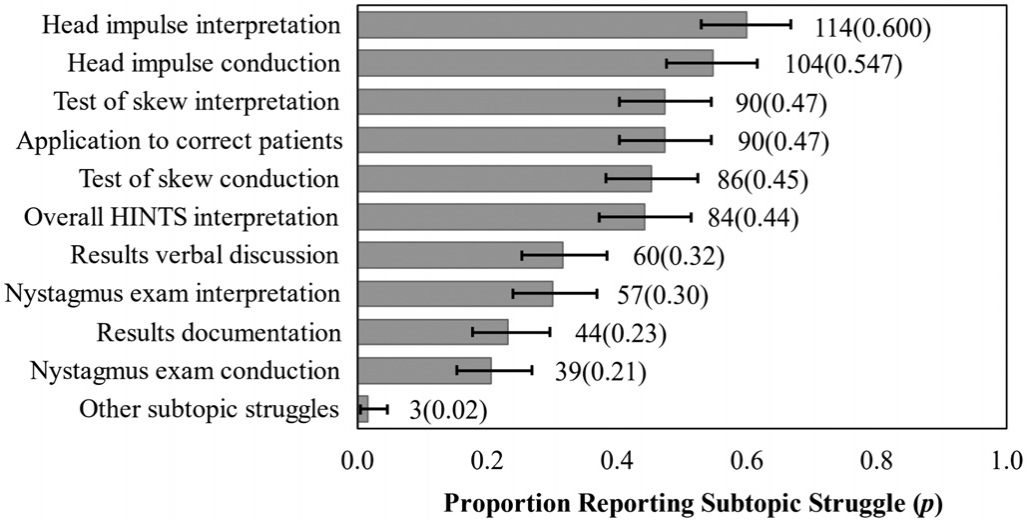
Frequency of residency program director-reported HINTS exam subtopic teaching struggles: 190 participants provided useable data on this item (item-level response rate 76.0%). Error bars represent the 95% confidence interval for these binomial proportion items using the Wilson statistic.

**Figure 3.**
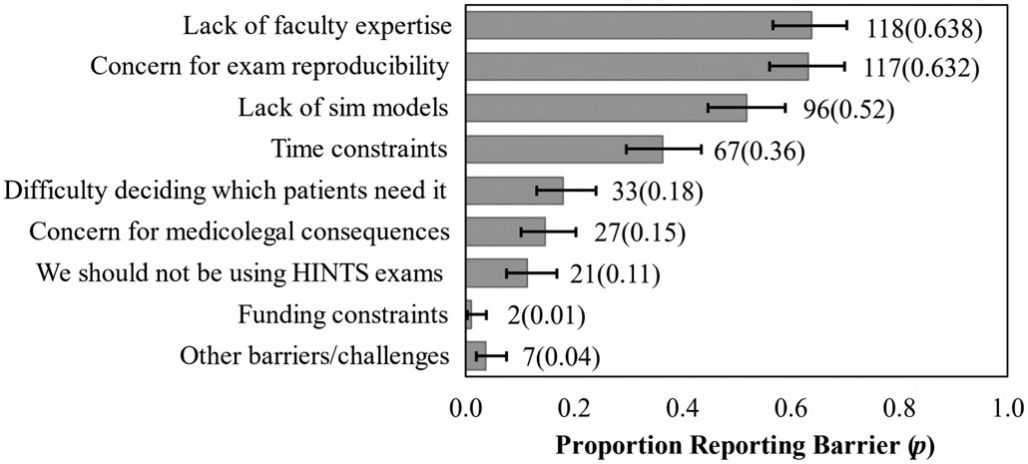
Frequency of residency program director-reported barriers to teaching the HINTS* examination. 185 participants provided useable data on this item (item-level response rate 74.0%). Error bars represent the 95% confidence interval for these binomial proportion items using the Wilson statistic. *HINTS, head impulse test, nystagmus, test of skew.

The most frequently cited HINTS subtopic teaching struggles centered around the HIT, test of skew, HINTS application to correct patients, and overall HINTS interpretation. See Figure 2. The most frequently cited HINTS teaching barriers centered around lack of faculty expertise, concern for poor HINTS exam reproducibility, and lack of resources. See Figure 3. Lastly, program leadership indicated that they would dedicate a mean of 2.0 hours/year (SD 1.3 hours/year) to implementing a formal, standardized HINTS exam curriculum if such a curriculum were widely available.

## DISCUSSION

Our findings may reflect underlying causes for the difficulties EPs have teaching and using the HINTS exam. Faculty members may hesitate to teach it if they exhibit discomfort with their own HINTS exam skills. In our findings, program leadership expressed lack of faculty expertise as an educational barrier, and they also perceived their residency graduates to be more competent with this skill than their faculty members (although this finding did not reach statistical significance). Additionally, program directors’ cited barrier of concern for poor HINTS exam reproducibility may point toward physicians’ innate desire for diagnostic certainty and the perception that the HINTS exam is fallible. Our respondents reported that HINTS is valuable to teach, but they less often reported HINTS offerings in conferences. The cited reasons for this discordance centered around lack of time, resources, and faculty expertise.

It has been shown in the original HINTS literature and subsequent research released from neurology and EM collaboration efforts that it is possible to effectively learn this skill,^13^ ^,^ ^21^ yet our results do not describe widespread skill acquisition among EPs. It is possible that educational collaboration between the specialties could positively impact EPs’ proficiency in the exam itself and in optimal educational methods. Regardless, our survey results support a desire to address the lack of faculty expertise. Much collaboration is already occurring, as evidenced by recent dizziness and vertigo literature authored by teams including members of both specialties,^21^ ^,^ ^28^ ^–^ ^30^ as well as research with mixed cohorts from both specialties.^1^

Our results show that simulation was perceived as one of the most effective modalities for HINTS education, but lack of simulation models was also cited as a top educational barrier. The HINTS education literature from neurology and neuro-subspecialties has proposed some innovative simulation adjuncts. For example, one study found that neurology trainees’ utilization of video-oculography (VOG) technology in simulation correlated with significant improvements in HIT performance.^31^ Two studies used virtual reality-enhanced manikin task trainers for HINTS simulation, demonstrating exam sensitivity and specificity improvements, including among EP cohorts.^32^ ^,^ ^33^ While such “partial task trainers” may have utility, our survey suggests they are not widely used or commercially available. The VOG devices are commercially available and have quality assurance (QA) features to assist examiners’ HIT performance via feedback on maneuver angles and velocities. A recent study used these QA features in EM resident HIT simulation and reported significant improvement in HIT maneuver performance.^34^

There are no published parameters from the neurology or subspecialty literature regarding the optimal HINTS curriculum training durations. However, a 2022 systematic review of HINTS and STANDING education reported on five institutions’ EM educational practices. They found wide curricular variability in didactic time (1–5 hours), workshop time (1–8 hours), neurology exposure (clinical rotations), and proctored exams (up to 15) over each resident’s duration in the program.^35^ Our participants indicated they would dedicate a mean of 2.0 hours/year to HINTS education, and over the course of a three- or four-year residency, this would be adequate time for the parameters described.^35^ Despite willingness to commit this time, other literature suggests that the exam application and maneuvers may be more complex than our specialty recognizes.^14^ As reflected in our results, program leadership perceived higher confidence than competence among graduates and faculty alike (although this did not reach statistical significance). This phenomenon—the Dunning-Kruger effect—is present in medicine, and existing literature suggests that assessments by examiners from multiple disciplines are required to ensure proficiency in such high-level skills.^36^ ^,^ ^37^ This would potentially add more time to a HINTS curriculum.

Our results contribute to a growing description of the HINTS educational modalities in use, but each modality has pros and cons beyond the training hours required. Clinical bedside teaching (the highest-rated modality in our survey) provides the highest-fidelity and real-life experience but is dependent on case convenience (dizzy/vertiginous patients presenting) and educator availability on shift. The opportunity cost of bedside training must be considered as well. The survey does not explore the hypothetical on-shift faculty time spent and associated opportunity cost, which would be a useful topic for future research.

Simulation tied for second place as the highest-rated HINTS educational modality. It mitigates the case convenience issue by providing on-demand patient cases in a controlled setting, but it also presents a faculty opportunity/cost issue by increasing training time in the simulation center. Hands-on skill simulation requires small-group or individual instruction, which uses more faculty time and the use of simulation models, and possibly other simulation adjuncts. Our survey did not ask about specific HINTS simulation equipment or techniques being used at EM residency programs in the US, but even if aggressive cost-of-implementation estimates are made, the return on investment would make HINTS educational initiatives financially worthwhile. The nationwide capital expenditures (specifically, VOG devices for simulation) cost about $9.76 million, which amounts to 1% of the estimated $1 billion/year spent on inappropriate CT imaging for patients with dizziness/vertigo in the US.^8^ The estimated national yearly cost after capital investment (specifically, the cost of faculty time) is about $331,883 in addition to costs for any equipment repair or new devices/adjuncts. See Appendix G for the cost-of-implementation analysis.^34^ ^,^ ^38^ ^–^ ^41^

Notably, HINTS manikin “partial task trainers” have been developed and tested, but none are widely available.^32^ ^,^ ^33^ The 2023 ACEP Clinical Policy recommended incorporation of technology such as Frenzel goggles and ocular tracking software in training.^20^ The VOG devices are commercially available for $12,000–40,000 per device and have shown promise in the simulation environment.^31^ ^,^ ^34^ ^,^ ^38^ ^,^ ^42^

To describe the effectiveness of many educational options (including those amenable to asynchronous and large-group sessions), we asked about several other modalities in addition to clinical bedside teaching and HINTS simulation. Online videos and videocasts were tied with simulation for the second highest-rated teaching modality among participants. Contrary to bedside and simulation teaching, this modality requires no faculty time or supervision and is free. Online HINTS educational videos can be used as an asynchronous supplement to clinical bedside teaching and simulation, but watching videos is a passive learning technique with no hands-on practice or opportunity for acquisition of muscle memory. However, recent studies suggest that achieving HINTS exam skills (particularly HIT skills) does require a hands-on component for motor skills acquisition.^34^

Overall, more time, effort, funding, and educational research could be targeted toward creating HINTS curricula and simulation modalities, and on making these resources widely available to improve EM residency HINTS educational options. The variability in our survey results shows that multiple education modalities are likely being employed across the residency training programs in the US but with some consensus about the most useful modalities. In such a situation where multiple modalities are being employed to the same end, further research toward development of a standardized training plan is needed.

## LIMITATIONS

To achieve adequate response rates from our survey, the length of the survey was limited at the recommendations of the expert pilot test group. Additionally, variability of the question design was employed to hold participants’ interest and increase response rates. As a result, some questions were asked in a binary “yes/no” format instead of Likert scales or rankings, potentially sacrificing some depth of response interpretation. Another concern with our survey design was response bias. While allowing questions on the survey to be left unanswered supports overall increased response rates, bias may have been introduced via respondent-allocated missing data. It is possible that program leaders who answered fewer questions had more passive opinions about the HINTS exam, exhibiting neutral response bias wherein, for example, they selected “neutral” or “no opinion” on classic Likert-scale questions. The opposite is also possible wherein the survey results are biased toward those in strongly in favor of or strongly against the HINTS exam (extreme response bias). Fortunately, our overall high response rates and wide variability of responses suggests these limitations are minimal.

Surveys were initially sent to EM residency program directors who had the option of either completing it themselves or assigning the responsibility to an associate program director, or to the faculty leader of the residency’s curricular content. There is, thus, a possibility that answers varied depending on the role of the survey-taker for each program, which was not recorded.

## CONCLUSION

Emergency medicine residency programs and medical educators should focus their HINTS educational priorities on development of a formalized curriculum with adequate resources. Programs will also need to address the barrier of lack of faculty expertise.

## Supplementary Information




