# Supplementary material for: Leadership Perceptions, Educational Struggles and Barriers, and Effective Modalities for Teaching Vertigo and the HINTS Exam: A National Survey of Emergency Medicine Residency Program Directors
Source: West J Emerg Med. 2024 Dec 31;26(1):70–7. doi: 10.5811/westjem.20787 (PMC11908532; doi:10.5811/westjem.20787)
Supplement: Supplementary file 1 [file wjem-26-70-s001.docx]

**Appendix A: Study Recruitment Message**

Dear Program Directors,

As part of the CORD MERC (Medical Education Research Certification) Program, we are conducting a national survey of EM residency programs assessing *Contemporary Practices on Dizziness and HINTS Exam Curricula*. We would be grateful if you can help this MERC research initiative by taking the survey. Please feel free to consult with whomever manages the resident didactic curriculum for your program. We are reaching out specifically to you, residency leadership, as you are both the most informed as to the current EM curriculum as well as future changes in our resident’s education. It takes 10 minutes and is anonymous, voluntary, and IRB-approved. For questions, concerns, or other comments, please reach out to our research team or the supervising IRB using the contact information below. Thank you!

**Appendix B: Survey Tool**

*Page 1: Survey Information and Consent*

Thank you for your participation in this research. This survey will be used to assess the current state of dizziness education and training for emergency medicine residency programs across the United States, particularly regarding the HINTS exam in the evaluation of acute vestibular syndrome.

This 10 minute survey is anonymous, voluntary, and IRB-approved. General program demographic information will be collected, data will be aggregated for analysis, and no email, IP addresses, or other identifying information will be collected. Your answers will be used only if you consent.

If you have any questions, comments, or concerns, please email the investigatory team ([redacted) or the Institutional Review Board ([redacted]).

1. Do you consent to have your answers used in this research study?
   1. Yes
   2. No

*Page 2: Hints Perceptions & Practices*

Definitions:

- The syndromic approach to dizziness is the organization of the complaint of dizziness into 4 different categories of dizziness based of the timing and triggers of the symptoms: triggered-acute vestibular syndrome vs. spontaneous-acute vestibular syndrome and triggered-episodic vestibular syndrome vs. spontaneous-episodic vestibular syndrome)
- The Acute Vestibular Syndrome (AVS) is defined as rapid onset (over seconds to hours) of vertigo, nausea/vomiting, and gait unsteadiness in association with head-motion intolerance and nystagmus, lasting days to weeks.
- The HINTS exam has been proposed as a tool to distinguish central from peripheral causes of AVS. The HINTS exam is a set of three structured bedside clinical assessments, including:
  - Head Impulse test of vestibulo-ocular reflex function
  - Nystagmus characterization in various gaze positions
  - Test of Skew for ocular alignment.

1. Do you believe the *HINTS exam* is a valuable skill for residency programs to teach to emergency medicine residents?
   1. Yes
   2. No
2. Does your conference curriculum include a formal educational session on evaluation of the “dizzy” patient?
   1. Yes
   2. No
   3. Other
3. Does your conference curriculum include a formal educational session on the *syndromic approach* to dizziness?
   1. Yes
   2. No
   3. Other
4. Do any members of your faculty provide beside/on-shift teaching of the *syndromic approach* to dizziness?
   1. Yes
   2. No
   3. Other
5. Does your conference curriculum include a formal educational session on the *HINTS exam*?
   1. Yes
   2. No
   3. Other
6. Do any members of your faculty provide beside/on-shift teaching of the *HINTS exam*?
   1. Yes
   2. No
   3. Other
7. Multimodal educational approaches are ideal for long-term retention and skill mastery. However, please rate your perceived relative effectiveness of the following educational modalities for teaching the HINTS exam. If you have never used a particular modality, select N/A.

| Exam element | ←Very ineffective Very effective→ | | | | | | | N/A |
| --- | --- | --- | --- | --- | --- | --- | --- | --- |
|  | 1 | 2 | 3 | 4 | 5 | 6 | 7 |  |
| Traditional didactic (lecture) | ◎ | ◎ | ◎ | ◎ | ◎ | ◎ | ◎ | ◎ |
| Small group discussion | ◎ | ◎ | ◎ | ◎ | ◎ | ◎ | ◎ | ◎ |
| Sim/simulation | ◎ | ◎ | ◎ | ◎ | ◎ | ◎ | ◎ | ◎ |
| Clinical bedside teaching | ◎ | ◎ | ◎ | ◎ | ◎ | ◎ | ◎ | ◎ |
| Journal club discussion | ◎ | ◎ | ◎ | ◎ | ◎ | ◎ | ◎ | ◎ |
| Grand rounds | ◎ | ◎ | ◎ | ◎ | ◎ | ◎ | ◎ | ◎ |
| Oral boards-style cases | ◎ | ◎ | ◎ | ◎ | ◎ | ◎ | ◎ | ◎ |
| Textbook reading | ◎ | ◎ | ◎ | ◎ | ◎ | ◎ | ◎ | ◎ |
| Journal article reading | ◎ | ◎ | ◎ | ◎ | ◎ | ◎ | ◎ | ◎ |
| Text-based asynchronous modules | ◎ | ◎ | ◎ | ◎ | ◎ | ◎ | ◎ | ◎ |
| Podcasts or audio recordings | ◎ | ◎ | ◎ | ◎ | ◎ | ◎ | ◎ | ◎ |
| Videocasts or online videos | ◎ | ◎ | ◎ | ◎ | ◎ | ◎ | ◎ | ◎ |
| Other (Free text - qualitative) | (enter free text) | | | | | | | |

1. To the best of your ability, please estimate the following.

|  | ←Least (worst) Most (best)→ | | | | | | | N/A |
| --- | --- | --- | --- | --- | --- | --- | --- | --- |
|  | 1 | 2 | 3 | 4 | 5 | 6 | 7 |  |
| Your faculty’s comfort level in performing and interpreting the HINTS exam? | ◎ | ◎ | ◎ | ◎ | ◎ | ◎ | ◎ | ◎ |
| Your faculty’s competence in performing and interpreting the HINTS exam? | ◎ | ◎ | ◎ | ◎ | ◎ | ◎ | ◎ | ◎ |
| Your residency graduates’ comfort level in performing and interpreting the HINTS exam? | ◎ | ◎ | ◎ | ◎ | ◎ | ◎ | ◎ | ◎ |
| Your residency graduates’ competence in performing and interpreting the HINTS exam? | ◎ | ◎ | ◎ | ◎ | ◎ | ◎ | ◎ | ◎ |

1. In your experience, which parts of the *HINTS exam* are a struggle for residency programs to teach? *(Select all that apply)*
   1. Application of the exam to the correct patients
   2. Proper conduction of the head impulse test (psychomotor skill)
   3. Proper interpretation of the head impulse test
   4. Proper conduction of the nystagmus exam (psychomotor skill)
   5. Proper interpretation of the nystagmus exam
   6. Proper conduction of the test of skew (psychomotor skill)
   7. Proper interpretation of the test of skew
   8. Proper interpretation of the overall HINTS exam results
   9. Proper verbal discussion of HINTS exam results (e.g., discussion with Neurology)
   10. Proper documentation of HINTS exam results
   11. Other (specify)
2. If a formal, standardized, high-quality *HINTS exam* curriculum was made available, how many hours per year could you dedicate to implementing it for your residents?

| *(Free text)* |
| --- |

1. What are the perceived barriers to teaching the HINTS exam to your residents?
   1. Time constraints
   2. Funding constraints
   3. Lack of HINTS exam expertise amongst faculty
   4. Lack of HINTS exam sim models (e.g., mannequin trainers)
   5. Concern for medicolegal consequences of using the HINTS exam
   6. Concern for poor reproducibility of HINTS exam results
   7. Difficulty deciding which patients should receive a HINTS exam.
   8. Emergency physicians should not use the HINTS exam at this time
   9. Other barriers/challenges (please specify - free text)
2. What have you done to address these barriers, and did it help?

| *(Free text)* |
| --- |

*Page 3: Hospital/Institution Characteristics*

Definitions:

- Comprehensive Stroke Center (CSC): Designed for those hospitals that have specific abilities to receive and treat the most complex stroke cases. The most demanding certification.
- Primary Stroke Center (PSC): Designed for hospitals that provide the critical elements of stroke care to achieve long-term success in improving outcomes.
- Thrombectomy-Capable Stroke Center (TSC): Designed for hospitals that are a Primary Stroke Center and also provides endovascular procedures and post-procedural care.
- Acute Stroke Ready Hospital (ASRH): Designed for hospitals or emergency centers that have a dedicated stroke-focused program.

1. What is the stroke center designation of your primary site (where your residents spend the most time)?
   1. Comprehensive Stroke Center (CSC)
   2. Primary Stroke Center (PSC)
   3. Thrombectomy-Capable Stroke Center (TSC)
   4. Acute Stroke Ready Hospital (ASRH)
   5. Not a stroke center
   6. Unsure
2. What modes of neurology consultation are available in the ED at your primary site?
   1. In-house or in-person consults
   2. Phone consults
   3. Telemedicine
   4. Teleneurology
   5. Tele-dizzy
   6. Other video-assisted virtual consults
   7. Other (specify)
3. For an ED patient with possible acute onset stroke, how many hours does it take to get a STAT MRI brain (with radiology read) from the time it is ordered?
   1. 0 to <2 hours
   2. 2 to <4 hours
   3. 4 to <6 hours
   4. 6 to <8 hours
   5. 8 to <10 hours
   6. >10 hours
   7. It is not possible for an ED patient

*Page 4: Residency Program Characteristics*

1. What is your residency program’s designation?
   1. University-Based
   2. Community-Based
   3. Community-Based/University Affiliated
   4. Military-Based
   5. Other (specify)
2. What is your residency program’s standard training duration?
   1. 3 years
   2. 4 years
3. Region of primary site
   1. New England (CT, MA, ME, NH, RI, VT)
   2. Mid Atlantic (NJ, NY, PA)
   3. East North Central (IL, IN, MI, OH, WI)
   4. West North Central (IA, KS, MN, MO, ND, NE, SD)
   5. South Atlantic (DC, DE, FL, GA, MD, NC, VA, WV)
   6. East South Central (AL, KY, MS, TN)
   7. West South Central (AS, LA, OK, TX)
   8. Mountain (AZ, CO, ID, MT, NM, NV, UT, WY)
   9. Pacific (AK, CA, HI, OR, WA)
   10. Territory (PR)
   11. Other (specify)
4. Geographic setting of primary site
   1. Urban
   2. Suburban
   3. Rural
   4. Other (specify)
5. For newer residency programs: has your program graduated at least one class of emergency medicine residents? (Specifically, residents already completed all requirements and finished working as of the date of this survey.)
   1. Yes
   2. No, not yet
   3. We are not a new program

**Appendix C: Program Enrollment Flowsheet**


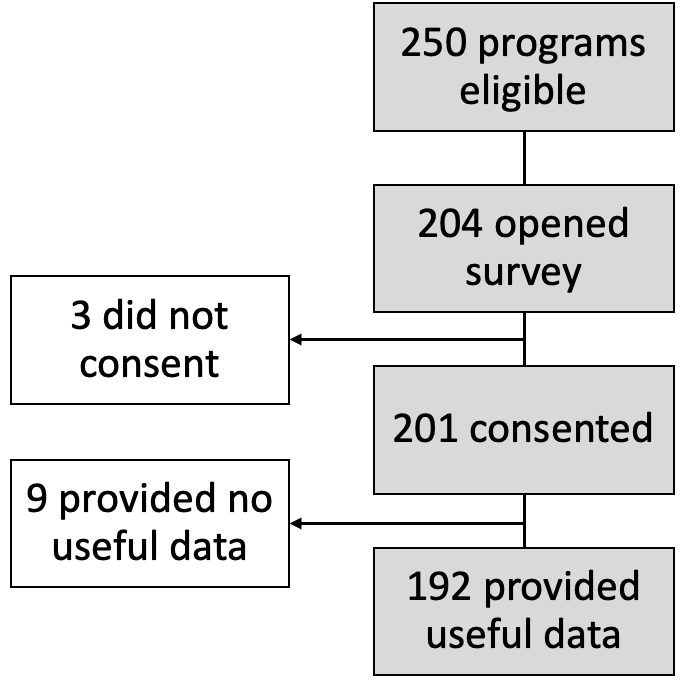


**Figure A1:** Participating residency program enrollment flowsheet.

**Appendix D: Participating Program Demographic Characteristics**

**Table A1.** Participating residency program demographic characteristics and comparison to the population of all 250 eligible residency programs.

| **Program Demographic** | **Study Sample, *n*(*p*)** | **Population, *n*(*p*)** |
| --- | --- | --- |
| Standard Training Length  3-year  4-year | 148(0.783)  41(0.217) | 199(0.796)  51(0.204) |
| Program Type  University  Community  University-Affiliated Community  Other (Military, County) | 85(0.450)  32(0.169)  66(0.349)  6(0.032) | 98(0.392)  50(0.200)  97(0.388)  5(0.020) |
| Region^a^  East North Central  East South Central  Mid Atlantic  Mountain  New England  Pacific  South Atlantic/Territory  West North Central  West South Central | 38(0.202)  7(0.037)  42(0.223)  9(0.048)  9(0.048)  18(0.096)  36(0.191)  9(0.048)  21(0.112) | 55(0.220)  10(0.040)  57(0.228)  10(0.040)  12(0.048)  23(0.092)  48(0.192)  11(0.044)  24(0.096) |

^a^States included in each region are shown in Appendix E.

**Appendix E: States and Territories Included in Regional Breakdown of Emergency Medicine Residency Programs**

- East North Central includes IL, IN, MI, OH, and WI
- East South Central includes AL, KY, MS, and TN
- Mid Atlantic includes NJ, NY, and PA
- Mountain includes AZ, CO, ID, MT, NM, NV, UT, and WY
- New England includes CT, MA, ME, NH, RI, and VT
- Pacific includes AK, CA, HI, OR, and WA.
- South Atlantic includes DC, DE, FL, GA, MD, NC, PR, VA, and WV
- West North Central includes IA, KS, MN, MO, ND, NE, and SD
- West South Central includes AR, LA, OK, and TX

**Appendix F: Program Leadership Perceptions of Residency Graduates and Faculty**

**Table A2.** Program leadership perceptions of residency graduate and faculty confidence and competence in performance and interpretation of the HINTS exam. Perceived confidence and competence were reported on a Likert scale from 1 (least) to 7 (most).

| **Item** | ***n*** | **Mean** | **95% CI** |
| --- | --- | --- | --- |
| Residency Graduates  Confident  Competence | 190  191 | 4.43  4.41 | 0.18  0.19 |
| Faculty Members  Confident  Competence | 190  189 | 4.16  4.15 | 0.20  0.20 |
